# Wideband Tympanometry and Pressurized Otoacoustic Emissions in Children with Surgical Excision of Palatine and/or Pharyngeal Tonsils

**DOI:** 10.3390/brainsci14060598

**Published:** 2024-06-14

**Authors:** Aline Buratti Sanches, Milaine Dominici Sanfins, Piotr Henryk Skarzynski, Magdalena Beata Skarżyńska, Henrique Costa Penatti, Caroline Donadon, Ingrid Pereira de Souza, Ingridy Vitoria da Silva, Maria Francisca Colella-Santos

**Affiliations:** 1 Faculty of Medical Sciences, State University of Campinas, Campinas 13083-887, São Paulo, Brazil; alinebsanches@gmail.com (A.B.S.); c101841@dac.unicamp.br (C.D.); i146483@dac.unicamp.br (I.P.d.S.); i174996@dac.unicamp.br (I.V.d.S.); mfcolell@unicamp.br (M.F.C.-S.); 2 Speech-Hearing-Language Department, Audiology Discipline, Universidade Federal de São Paulo, São Paulo 04023-062, São Paulo, Brazil; 3 Department of Teleaudiology and Screening, Institute of Physiology and Pathology of Hearing, 05-830 Kajetany/Warsaw, Poland; p.skarzynski@ifps.org.pl; 4 Department of Otorhinolaryngology Hearing, Center of Hearing and Speech Medincus, 05-830 Kajetany, Poland; m.skarzynska@csim.pl; 5 Department of Clinical Trials, Institute of Sensory Organs, 05-830 Kajetany, Poland; 6 Department of Hearing, World Hearing Center, Institute of Physiology and Pathology of Hearing, 05-830 Kajetany, Poland; 7 Department of Pharmacotherapy and Pharmaceutical Care, Faculty of Pharmacy, Medical University of Warsaw, 02-097 Warsaw, Poland; 8 Department of Otolaryngology, Ambulatory of Medical Specialties, Santa Bárbara D’Oeste 13450-000, São Paulo, Brazil; hpenatti@hrp.unicamp.br

**Keywords:** children, hearing, palatine tonsil, pharyngeal tonsil, surgery

## Abstract

Palatine and pharyngeal tonsil hypertrophy may lead to dysfunction of the auditory tube due to a propensity for infection, potentially giving rise to otitis media. This is a quantitative and longitudinal study, developed from 2019 to 2021, at the State University of Campinas (UNICAMP). The studied sample comprised 15 participants aged 5 to 12 years (mean 7.9 years), 12 male and 3 female, arranged into two groups: children diagnosed with pharyngeal and/or palatine tonsil hypertrophy who were candidates for surgery (G1), and children who were later evaluated after surgery (G2). As part of the test, an otoscopy and measurements of logoaudiometry, pure-tone threshold audiometry, wideband tympanometry (ambient and peak pressure), and otoacoustic emissions (TEOAEs and DPOAEs, both at ambient and peak pressure) were all performed. There were statistically significant differences between phases in pure-tone audiometry, in terms of 226 Hz tympanometry, wideband tympanometry in peak pressure conditions, in the amplitude measurement TEOAEs in both pressure conditions, in DPOAEs in ambient pressure conditions, and in the signal/noise measurement in both pressures in DPOAEs. Overall, it was found that hearing tests were different for subjects with palatine and pharyngeal tonsil hypertrophy compared to the post-surgical group.

## 1. Introduction

The auditory system is made up of sensory structures and central connections, consisting of a peripheral and central portion. The peripheral system comprises the structures of the external ear, middle ear, inner ear, and vestibulocochlear nerve, located in the temporal region of the skull. These structures are responsible for the reception, detection, conduction, and transduction of acoustic signals into neuroelectric impulses. The central auditory system comprises the auditory pathways, brain stem, and cortical areas, which are responsible for analyzing and interpreting sound stimuli and the functions of central auditory processing [1].

Changes in the peripheral auditory system can occur due to several factors. One of them is hypertrophy of the pharyngeal and palatine tonsils, which can obstruct the upper airways, causing oral breathing and poor Eustachian tube function [2]. The immunological function of the pharyngeal and palatine tonsils leads to their rapid growth during the first years of life, reaching their maximum size at age 6 for the pharyngeal tonsils and puberty for the palatine tonsils. After this period, involution normally occurs through increased production of fibrous tissue and atrophy of fatty tissue, which generally occurs around 8 to 10 years of age and during adulthood, respectively [3]. However, hypertrophic adenoids and tonsils do not atrophy normally [4].

As they represent the body’s first contact with microorganisms and substances, the tonsils are commonly the site of a range of pathological processes, mainly fighting off infection, and this occurs mostly between the ages of 2 and 5 years [5]. In cases where there is chronic inflammation of these structures, surgical removal is recommended. Worldwide, adenotonsillectomy is one of the most commonly performed surgical procedures and the most common form of otorhinolaryngological surgery, especially in children [6].

Because tonsil hypertrophy is so common in children and can have serious effects, it is very important to perform objective hearing tests on this population. This is because the condition may be linked to otitis media, hearing loss, and central auditory processing disorders, all of which can hurt the development of children.

To detect changes in hearing, audiological assessments are required. One procedure is acoustic immittance measurement, which can evaluate the function and integrity of the middle ear. Tympanometry is a dynamic way to measure acoustic immittance that checks how mobile the tympanic-ossicular chain system is when air pressure changes in the external acoustic cavity (EAC) [7,8,9]. Conventionally, tympanometry is performed using a single frequency, commonly 226 Hz. More recently, wideband tympanometry (WBT) has been used, as it measures the efficiency of the middle ear in transmitting sound at frequencies from 226 to 8000 Hz. WBT allows pressurized and non-pressurized measurements to be performed, allowing evaluation in patients with a ventilation tube or perforated tympanic membrane [10]. WBT can also find out the values of reflectance and absorbance, which change depending on the health of the tympanic-ossicular chain. This gives us more details about the health of the middle ear [11].

Otoacoustic emissions (OAE), whether transient (TEOAE) or distortion product (DPOAE), are electroacoustic tests that evaluate cochlear function. The outer hair cells are connected to the normal functioning of the cochlear amplifier, and OAEs are by-products of those mechanisms. They can change depending on the type of stimulus and the sex of the person [12]. Abnormal OAEs can be a sign of hearing loss due to damaged structures in the inner ear or various pathologies of the middle ear, such as perforation of the eardrum, otitis media, and the presence of impacted cerumen [13].

It is also worth highlighting that the conductive structures, including the pinna, the external auditory canal, the tympanic membrane, and the middle ear, are essentially well developed at birth [14]. Initially, the neonate ear canal is relatively flaccid and prolapsed, but it begins ossifying prenatally and continues throughout the first years of life, while the cochlea is mature and adult from birth [15]. In this way, the life stages of children and adolescents do not have an impact on the responses of WBT and OAE.

Hearing assessments using complementary methods can help diagnose problems and establish remedial actions. They are also useful to monitor subjects after they have undergone surgical procedures. The present study focused on children before and after surgery for removal of pharyngeal and/or palatine tonsils. We evaluated the peripheral auditory system of these children through broadband tympanometry, TEOAEs, and DPOAEs under pressurized and non-pressurized conditions.

This study had the main objective of analyzing the peripheral auditory system of children with surgical indications for removal of the pharyngeal and/or palatine tonsils before and after surgery. The second goal was to look at the results of tests that measured tympanometry across a wide frequency range and the transient and distortion product of otoacoustic emissions, taking into account the moments before and after the tests.

## 2. Materials and Methods

### 2.1. Ethics

This is a quantitative, comparative, and descriptive study that used a cross-sectional and longitudinal approach. The project was approved by the Research Ethics Committee of the State University of Campinas under opinion 3,753,188. Data were collected between the years 2019 and 2021. The subjects were invited to voluntarily participate in the research and were accompanied by a guardian. A free and informed consent form was signed by the guardian, and an assent form by the minor.

### 2.2. Participants

The initial proposal was for 59 individuals in the pre-operative group to undergo re-evaluation after the surgical procedure; however, due to the pandemic, a large number of patients did not agree to return for the re-evaluation process. However, it should also be noted that isolation due to COVID had positive impacts on cases of tonsil hypertrophy. A study carried out on children who were awaiting adenotonsillectomy surgery identified that there was an improvement in symptoms during quarantine, demonstrating that confinement can have a positive impact on specific diseases derived from early socialization, in a way that causes changes in medical and surgical therapeutic indications [16]. Another study suggested that the prevalence of OME has returned to pre-lockdown levels, and that interrupting day care center attendance for a two-month period could be effective in resolving most cases of chronic OME [17].

The study sample consisted of 15 children between 5 and 12 years old (mean 7.9 years), comprising 12 males and 3 females. Considering the phases in which they were evaluated, they were grouped into two groups: G1, the pre-operative study group comprising children whose otorhinolaryngologists said they needed surgery to remove their palatine and/or pharyngeal tonsils, and G2, the post-operative study group comprising children from G1 who were re-evaluated after the surgery.

The children who made up G1 came from an otorhinolaryngology outpatient clinic, where the researcher attended medical appointments and invited children to participate in this study.

G2 was made up of the subjects from G1 who had undergone surgery to remove their tonsils.

The data were obtained between the end of 2019 and the end of 2021, a period in which we went through the pandemic, and both research and outpatient activities were suspended for a year.

### 2.3. Inclusion Criteria

The individuals who qualified for G1 had to have undergone an otorhinolaryngological examination, have a surgical indication for palatine and/or pharyngeal tonsil hypertrophy, and be free of any complaints, neurological changes, or cognitive deficits (as reported by the parent and supported by medical records). For G2, the criteria were: having been evaluated preoperatively; and having undergone surgery to remove the pharyngeal and/or palatine tonsils in the previous 3 to 6 months. All children presented results within normal limits for speech audiometry.

### 2.4. Exclusion Criteria

The exclusion criteria for the present study were as follows: children aged younger than 5 years or older than 12 years; lack of authorization from the person responsible for the child; and children with tympanic membrane perforation, cholesteatoma, tympanosclerosis, aural atresia, craniofacial anomalies, cleft palate, genetic syndromes, neurological changes, or cognitive deficits.

### 2.5. Procedures

An otorhinolaryngological exam, a meatoscopy, speech audiometry, pure-tone audiometry, broadband tympanometry at ambient pressure (AP) and peak pressure (PP), an investigation of acoustic reflexes, and tests of transient otoacoustic emissions (TEOAEs) and distortion product otoacoustic emissions (DPOAEs) at ambient pressure (AP) and peak pressure (PP) were all performed on the participants.

The otorhinolaryngological evaluations were carried out at the Ambulatory of Medical Specialties of Santa Bárbara D’Oeste, and the audiological evaluations were carried out at the Audiology Laboratories of the Department of Human Development and Rehabilitation/Faculty of Medical Sciences of the State University of Campinas.

Hearing assessments were carried out with the necessary equipment in the institution’s audiology laboratories and were performed in a single day.

#### 2.5.1. Otorhinolaryngological Evaluation

The same otorhinolaryngologist performed all of the otorhinolaryngological evaluations, which included an otoscopy, rhinoscopy, and otoscopy physical examination after reviewing the patient’s clinical history. When enlargement of the tonsils was observed on physical examination, a classification of 1 to 4 was given according to the degree of hypertrophy and obstruction of the oropharynx (following the classification of Brodsky [18]). The degrees of turbinate hypertrophy were also observed, as were any craniofacial characteristics, such as adenoid facies or maxillary atresia. For the diagnosis of pharyngeal tonsil hypertrophy, flexible nasofibroscopy or cavum radiography were performed as necessary. For surgical indications, the following criteria were used: loud and persistent snoring, observed apnea, dysphagia due to tonsillar hypertrophy, oral breathing that does not get better with medical treatment, or recurrent tonsillitis or rhinosinusitis (according to the Paradise criteria [19]).

#### 2.5.2. Otoscopy

All children underwent a meatoscopy to check for any impediments to carrying out audiological assessments. Only patients who had no changes in their otoscopy during their initial visit with an otorhinolaryngologist were eligible to participate in the studies. If there was any change, the patient was excluded from the study.

#### 2.5.3. Pure-Tone Audiometry

A soundproof booth (Redusom) using an audiometer (model AC40, manufacturer Interacoustics, Middelfart, Denmark) and supra-aural headphones (model TDH 49, manufacturer Interacoustics, Middelfart, Denmark) was used to test frequencies from 250 to 8000 Hz with pure tones to find the hearing threshold. The normality criteria adopted were those of Northern and Downs: hearing thresholds ≤ 15 dBHL when the frequencies 500, 1000, and 2000 Hz were averaged [20].

#### 2.5.4. Logoaudiometry

Logoaudiometry consisted of a speech recognition threshold (SRT) and a speech recognition percentage index (SRPI), both carried out in a soundproof booth and with the same equipment as used in tonal audiometry. The SRF sought to confirm the tonal thresholds obtained in audiometry by obtaining the minimum level of intensity at which the subject could correctly repeat 50% of the words presented. The SRPI was a measure of speech intelligibility at an intensity of 40 dB, as determined by correct answers to a list of monosyllables. Values of 88% to 100% on SRPI were adopted as a normality criterion [21].

#### 2.5.5. Wideband Tympanometry (Ambient and Peak Pressure)

In order to test for conditions of the middle ear, broadband tympanometry was performed with Interacoustics Titan equipment, connected via a USB cable to a portable computer, probe, and earpiece. With the probe inserted into the EAC, a click stimulus was presented at 96 dB SPL and a pressure that swept from +200 to −300 daPa depending on the performance condition. The microphone in the probe picked up the stimulus that came back from the tympanic membrane. The software then calculated the acoustic absorbance by looking at the relationship between the stimulus and the acoustic absorbance. This created a three-dimensional graph with frequency (226–8000 Hz), pressure, and acoustic absorbance as its axes.

There were 107 separate frequencies on the absorbance curve, spanning from 226 to 8000 Hz. For this study, 17 frequencies were chosen at random, based on results from other research [22,23,24,25]. These frequencies were 226, 257, 324, 408, 500, 630, 794, 1000, 1260, 1587, 2000, 2520, 3175, 4000, 5040, 6350, and 8000 Hz. In each ear, two scans were performed: one at ambient pressure (AP, 0 daPa) and the other at peak pressure (PP). A measurement was taken at peak pressure (the point where the two-dimensional broadband tympanometry graph [23] showed the highest specific absorbance for each subject). According to the equipment’s shadow curve of normality, the acoustic absorbance curve was either classified as normal or altered.

The mobility of the tympanic-ossicular chain system was examined by measuring the tympanometric curve at a frequency of 226 Hz while air pressure was changed in the EAC. The reference values used were the peak compliance at atmospheric pressure (0 daPa) for an equivalent volume of 0.3 to 1.3 mL. The acoustic reflex was tested ipsilaterally and contralaterally at frequencies of 500, 1000, 2000, 3000, and 4000 Hz. A reflex at 70 to 100 dB above the threshold for pure tone from 500 to 4000 Hz was expected [7].

#### 2.5.6. TEOAEs and DPOAEs (Ambient and Peak Pressure)

TEOAEs and DPOAEs were performed to record the activity of outer hair cells. The equipment used to capture emissions was the same equipment as used in wideband tympanometry. Emissions were obtained at peak pressure (pressurized) and ambient pressure (non-pressurized) conditions.

TEOAEs were registered in response to 300 stimuli at 83 dBpeSPL over the frequency range of 1000 to 5000 Hz. To show that TEOAEs were present, a signal-to-noise ratio of at least 6 dBSPL was used for four frequency bands in a row, with an overall reproducibility of at least 90% and probe stability of at least 90%. We recorded DPOAEs in the DP-gram mode while two stimuli, f1 and f2, were shown at the same time. The ratio of f2 to f1 was 1.22, and the dBSPL levels were 65/55 over the frequency range of 500 to 10,000 Hz. The criterion used to indicate the occurrence of DPOAEs was a signal/noise ratio ≥ 6 dB and an amplitude above −10 dB SPL [26,27].

### 2.6. Statistical Analyses

A statistician examined the results. SPSS V20, Minitab 16, and Excel Office 2010 were used for statistical analysis.

The significance level adopted was 5% (*p* < 0.05). Parametric statistical tests were used.

To compare the two groups in the distribution of qualitative variables, a chi-square test was used.

To compare the qualitative factors of G1 and G2, a chi-square test was used, and for the quantitative factors, a paired Student’s *t*-test was used.

To compare the results between the groups in different pressure situations (peak pressure vs. ambient pressure), a paired Student’s *t*-test was used.

The results are shown in the accompanying tables. In the tables, statistically significant *p*-values are shown with an asterisk symbol (*). The symbol ‘x’ indicates that it was not possible to use the statistics in the data set.

## 3. Results

### 3.1. Sample Characterization

The sample consisted of 15 children, divided into two groups: (i) a study group pre-surgery (G1) and (ii) a study group post-surgery (G2). From the otorhinolaryngological exam, a surgical indication was found for all of the subjects.

There were 12 children who had both their pharyngeal and palatine tonsils removed at the same time, and 3 children who only had their pharyngeal tonsils removed (adenoid).

Table 1 shows the subjects distributed by age, sex, and structures removed in surgery.

Statistical analysis was performed examining the results considering the ear, and there was no significant difference; thus, the researchers chose to group the ears, so *n* represented 30 ears in the following tests.

### 3.2. Pure-Tone Audiometry

Table 2 shows the comparison between groups of auditory thresholds from pure-tone audiometry at frequencies from 250 to 8000 Hz, in the pre- and post-surgical phases. There was a statistically significant difference between phases at frequencies of 1000 Hz, 2000 Hz, and 3000 Hz, with better auditory thresholds in the G2 (post-surgery phase).

### 3.3. Tympanometry (226 Hz and Wideband)

Table 3 shows a comparison of the phases in relation to the classification (normal or altered) of results, following the criteria of each test, pure tone audiometry, tympanometry, and research on ipsi and contralateral acoustic reflexes. There was a statistically significant difference between the groups in terms of 226 Hz tympanometry, with G2 exhibiting better results.

In the AP condition, there was no significant difference between groups, while in the PP condition (Table 4), there was a significant difference between the groups at frequencies from 500 to 749 Hz.

There was no significant difference between the phases in the classification (normal or altered) of the absorbance curve, both for the peak pressure and ambient pressure conditions.

### 3.4. TEOAEs and DPOAEs

Table 5 shows the amplitude measurement of transient evoked otoacoustic emissions (TEOAEs) between frequencies from 1000 Hz to 5000 Hz in the peak pressure and ambient pressure conditions in both groups. The results indicate that there was a significant difference between the groups in the amplitude measurement, only for the frequency of 1000 Hz in PP and AP.

There was no significant difference between groups for the signal/noise ratio measurement in both pressure conditions in TEOAEs.

Table 6 shows a comparison between groups in terms of amplitude of otoacoustic emissions by distortion product in peak pressure and ambient pressure conditions in both groups (paired Student’s *t*-test).

This table shows the amplitude measurement of otoacoustic emissions by distortion product (DPOAEs) between frequencies from 1000 Hz to 5000 Hz in peak pressure and ambient pressure conditions in both groups. The results indicate that there was a significant difference between the groups in the amplitude measurement, only for the frequency of 8000 Hz in AP.

Table 7 shows a comparison between groups in terms of signal/noise of otoacoustic emissions by distortion product in peak pressure and ambient pressure conditions in both groups (paired Student’s *t*-test).

This table shows the signal/noise measurement of otoacoustic emissions by distortion product (DPOAEs) between frequencies from 1000 Hz to 5000 Hz in peak pressure and ambient pressure conditions in both groups. The results indicate that there was a significant difference between the groups in the signal/noise measurement for frequencies from 1000 Hz to 3000 Hz in PP, and for frequencies from 1000 Hz to 4000 Hz in AP.

## 4. Discussion

The present study aimed to understand the differences in how the peripheral auditory system functions in children with hypertrophy of the pharyngeal and/or palatine tonsils. We tested these children both before and after the removal of these structures using 226 Hz and wideband tympanometry, pure-tone audiometry, and otoacoustic emissions. To date, few studies have been carried out on this population using different assessment methods.

### 4.1. Pure-Tone Audiometry

The main objective of audiological assessment is to determine the integrity of the auditory system as well as identify the type, degree, and configuration of hearing loss in each ear. Tonal threshold audiometry is fundamental to audiological diagnosis and is the gold standard test for evaluating hearing [28].

Comparisons between G1 and G2 showed that G1 had higher (or poorer) tonal thresholds for frequencies from 1000 to 3000 Hz. The improvement in pure-tone audiometry for G2 was probably due to the surgical procedure, corroborating previous studies that studied hearing loss in patients following otorhinolaryngological treatment, including cases of adenotonsillar hypertrophy [29,30]. However, both groups presented mean and median hearing thresholds within normal limits at all frequencies. It should be noted that good hearing sensitivity at medium and high frequencies is important for understanding and acquiring speech, and sensory deprivation can harm child development.

### 4.2. Tympanometry (226 Hz and Wideband)

Tympanometry is an objective and rapid test that investigates the integrity of the tympanic-ossicular system and helps identify middle ear changes. These occur mainly in schoolchildren, such as the children in the present study [7,31].

Tympanometry using a 226 Hz probe showed no statistically significant difference between the groups. These data diverge from the literature, where it is reported that changes in the adenoid and/or palatine tonsils tend to produce negative middle ear pressures, with values ranging from −100 to −400 daPa. This shift in the tympanometric curve is probably related to mechanical obstruction of the auditory tube [32,33]. Abdel and Tabook reported a highly significant relationship between adenoid hypertrophy and the presence of a type B tympanogram; they also reported a high correlation between adenoid size and the incidence of otitis media with effusion [34].

When comparing subjects in the pre- and post-surgical phases, there was a statistical difference in tympanometry, in which the normality index rose from 86.7% in the pre-surgical phase to 100% in the post-surgical phase. The altered tympanometric curves obtained in the pre-surgical phase were classified as two type B curves and two type C curves. There was no significant difference between the phases for compliance and pressure. However, when observing the descriptive pressure values, we identified that in the comparison between the groups, the subjects in pre-surgical phase showed a wide variation in negative pressure, which recovered in the post-surgical phase. Bluestone et al. indicated that enlarged adenoids can lead to mechanical obstruction of the auditory tube, leading to air absorption and negative intratympanic pressure [32].

Thus, our study demonstrates that children with adetonsillar hypertrophy presented tympanometric changes, indicative of otitis media and tubal dysfunction. This result is in agreement with the study by Bianchini et al., which concluded that patients with hypertrophy are more susceptible to tympanometric changes [29]. According to Salvinelli et al., surgery for chronic nasal obstruction significantly improves tubal function and middle ear ventilation at least one month after the surgical procedure [35]. Therefore, our result is in line with an improvement in tympanometric findings after a period of 3–6 months following the surgical procedure.

When comparing acoustic reflexes in the pre- and post-phases (Table 3), we found no statistical difference, but it was possible to observe an improvement in responses post-operatively. The absence of the acoustic reflex is an indication of changes in the middle ear; however, due to the high complexity of the neural mechanism involved, the presence of increased threshold values and/or the absence of the acoustic reflex in individuals with tonal auditory thresholds within the standard normality may be associated with the presence of changes in language and auditory processing [36].

When analyzing the measurement of acoustic absorbance in the pre- and post-surgery phases, the results indicated a significant difference for the frequencies of 500, 630, and 749 Hz, in which the average post-surgery absorbance was increased; that is, the subjects showed greater absorbance after surgery for all frequencies. It was observed that there was no significant difference between the phases in the classification of the absorbance curve in both the peak pressure and ambient pressure conditions.

Our findings are in line with a literature review performed by Hunter and colleagues that confirmed that WBT has the potential to be a better diagnostic tool than traditional tympanometry because it can precisely measure how the middle ear receives, absorbs, and sends sound energy at different frequencies [37].

There are no previous studies of WBT in children with hypertrophy, and so the present work adds important information in this area. It is also worth noting that WBT can be useful in monitoring different stages of treatment, particularly before and after a surgical procedure, providing a marker of conditions and improvements in the middle ear.

### 4.3. TEOAEs and DPOAEs

OAEs aim to evaluate the functioning of the cochlea via measurements of outer hair cell responses, and can be used to help diagnose central auditory impairment. The most clinically useful otoacoustic emissions are transient stimulus otoacoustic emissions (TEOAEs) and distortion product otoacoustic emissions (DPOAEs) [38].

Combining OAE testing with tympanometry is appropriate for identifying middle ear changes [39]. The presence of middle ear abnormalities, such as in cases of otosclerosis and otitis media, affects OAE amplitudes, as the integrity of the middle ear and tympanic membrane are crucial for detecting outer hair cell responses.

Studies have shown that ambient pressure measurements may have sufficient accuracy for use in some hearing screening applications, but the pressurized condition provides additional information that may be useful for diagnostic applications [40].

### 4.4. Limitations and Future Research

Early hearing screening of children can help avoid possible adverse impacts on their global development. It is therefore suggested that studies in the area be continued, and that a hearing assessment protocol for all children who present clinical signs of tonsil hypertrophy be implemented.

The present study was carried out during the SARS-COVID-19 pandemic. Collections were initially suspended and then gradually resumed after making services more flexible and with the authorization of the institution. Unfortunately, even after introducing more flexible testing, many parents and guardians chose not to continue with the research, resulting in a reduced number of cases for follow-up.

Therefore, we are continuing our work, aiming to deepen our knowledge of the effect of hypertrophy of the pharyngeal and/or palatine tonsils on auditory function.

## 5. Conclusions

This study used an updated comprehensive middle ear evaluation methodology that included wideband tympanometry, which allowed for a thorough assessment of middle ear status. Using a variety of peripheral auditory assessments, we demonstrated that children with pharyngeal and/or palatine tonsil hypertrophy had improved auditory responses after surgery. Our findings support the implementation of wideband tympanometry as a standard in pediatric audiology.

## Figures and Tables

**Table 1:** Characterization of 15 subjects before surgery by age, sex, and structures removed in surgery.

	Age (Years)	5		6		7		8		9		10		11		12	
	Sex	F	M	F	M	F	M	F	M	F	M	F	M	F	M	F	M
Structures removed in surgery	Pharyngeal and palatine tonsils	0	2	0	1	0	1	1	3	0	2	0	1	1	0	0	0
	Pharyngeal tonsils	0	0	0	0	0	1	1	0	0	0	0	1	0	0	0	0

Legend: F, female; M, male.

**Table 2:** Comparison between groups in terms of pure-tone thresholds (paired Student’s *t*-test).

Frequency (Hz)	Group	*n*	Mean (dB HL)	Median	SD	Min	Max	*p*-Value
250	G1	30	8.17	10	5.94	−5	20	0.889
	G2	30	8.00	10	4.66	0	20	
500	G1	30	6.67	5	5.14	−5	15	0.086
	G2	30	5.00	5	4.35	−5	15	
1000	G1	30	4.33	5	4.50	−5	15	* 0.014
	G2	30	1.83	0	3.07	−5	10	
2000	G1	30	4.83	5	4.45	−5	15	* 0.037
	G2	30	2.83	5	3.39	−5	10	
3000	G1	30	5.17	5	3.82	0	10	* <0.001
	G2	30	1.83	0	3.59	−5	10	
4000	G1	30	3.17	5	4.64	−5	15	0.119
	G2	30	1.67	0	5.14	−5	15	
6000	G1	30	5.67	5	6.12	−10	15	0.202
	G2	30	7.33	5	6.12	0	20	
8000	G1	30	4.67	5	5.71	−5	20	0.778
	G2	30	5.00	5	6.57	−10	15	

Legend: G1, group pre-surgery; G2, group post-surgery; *n*, number of subjects; SD, standard deviation; Min, minimum; Max, maximum; *p*-value, * *p* ≤ 0.05.

**Table 3:** Comparison of subjects in relation classifications (normal or altered) of the results of pure-tone audiometry, and acoustic reflex research, in the pre- and post-surgical phases (chi-square test).

Test	Classification	G1		G2		Total		*p*-Value
		*n*	%	*n*	%	*n*	%	
Pure tone audiometry	Altered	0	0%	0	0%	0	0%	-x-
	Normal	30	100%	30	100%	60	100%	
Tympanometry	Altered	4	13.3%	0	0%	4	6.7%	* 0.038
	Normal	26	86.7%	30	100%	56	93.3%	
Reflex IPSI 500	Absent	7	23.3%	5	16.7%	12	20.0%	0.519
	Present	23	76.7%	25	83.3%	48	80.0%	
Reflex IPSI 1000	Absent	5	16.7%	3	10.0%	8	13.3%	0.448
	Present	25	83.3%	27	90.0%	52	86.7%	
Reflex IPSI 2000	Absent	6	20.0%	3	10.0%	9	15.0%	0.278
	Present	24	80.0%	27	90.0%	51	85.0%	
Reflex IPSI 4000	Absent	11	36.7%	8	26.7%	19	31.7%	0.405
	Present	19	63.3%	22	73.3%	41	68.3%	
Reflex CONTRA 500	Absent	21	70.0%	19	63.3%	40	66.7%	0.584
	Present	9	30.0%	11	36.7%	20	33.3%	
Reflex CONTRA 1000	Absent	20	66.7%	20	66.7%	40	66.7%	1.000
	Present	10	33.3%	10	33.3%	20	33.3%	
Reflex CONTRA 2000	Absent	10	33.3%	12	40.0%	22	36.7%	0.592
	Present	20	66.7%	18	60.0%	38	63.3%	
Reflex CONTRA 3000	Absent	14	51.9%	13	48.1%	27	50.0%	0.785
	Present	13	48.1%	14	51.9%	27	50.0%	
Reflex CONTRA 4000	Absent	13	43.3%	14	46.7%	27	45.0%	0.795
	Present	17	56.7%	16	53.3%	33	55.0%	

Legend: G1, group pre-surgery; G2, group post-surgery; *n*, number of ears; %, percentage; *p*-value, * *p* ≤ 0.05; -x-, was not possible to use the statistics in the data set.

**Table 4:** Comparison between groups in terms of absorbance at peak pressure from wideband tympanometry (paired Student’s *t*-test).

Frequency (Hz)	Group	*n*	Mean (dB HL)	Median	SD	Min	Max	*p*-Value
226 PP	G1	30	0.101	0.094	0.050	0.001	0.212	0.057
	G2	30	0.121	0.116	0.056	0.047	0.259	
257 PP	G1	30	0.107	0.099	0.052	0.003	0.222	0.060
	G2	30	0.128	0.123	0.059	0.050	0.273	
324 PP	G1	30	0.136	0.119	0.062	0.029	0.288	0.055
	G2	30	0.162	0.152	0.075	0.055	0.345	
408 PP	G1	30	0.187	0.155	0.078	0.088	0.375	0.055
	G2	30	0.221	0.209	0.101	0.069	0.434	
500 PP	G1	30	0.240	0.203	0.097	0.103	0.451	* 0.031
	G2	30	0.284	0.264	0.125	0.093	0.512	
630 PP	G1	30	0.338	0.307	0.131	0.135	0.670	* 0.047
	G2	30	0.390	0.382	0.155	0.134	0.670	
749 PP	G1	30	0.440	0.420	0.155	0.186	0.714	* 0.046
	G2	30	0.498	0.486	0.176	0.196	0.766	
1000 PP	G1	30	0.598	0.603	0.169	0.249	0.862	0.159
	G2	30	0.639	0.637	0.153	0.389	0.933	
1260 PP	G1	30	0.653	0.684	0.131	0.260	0.840	0.644
	G2	30	0.667	0.683	0.128	0.444	0.978	
1587 PP	G1	30	0.667	0.697	0.127	0.351	0.846	0.729
	G2	30	0.676	0.733	0.149	0.352	0.909	
2000 PP	G1	30	0.652	0.654	0.168	0.331	0.932	0.973
	G2	30	0.653	0.726	0.221	0.085	0.947	
2520 PP	G1	30	0.729	0.733	0.204	0.324	0.992	0.590
	G2	30	0.752	0.790	0.196	0.343	0.989	
3175 PP	G1	30	0.767	0.798	0.176	0.348	0.989	0.629
	G2	30	0.744	0.784	0.203	0.303	0.990	
4000 PP	G1	30	0.728	0.763	0.174	0.291	0.949	0.302
	G2	30	0.677	0.725	0.166	0.250	0.968	
5040 PP	G1	30	0.561	0.551	0.177	0.272	0.947	0.754
	G2	30	0.545	0.555	0.186	0.199	0.880	
6350 PP	G1	30	0.311	0.283	0.116	0.161	0.545	0.869
	G2	30	0.305	0.284	0.146	0.104	0.670	
8000 PP	G1	30	0.230	0.187	0.132	0.066	0.584	0.450
	G2	30	0.258	0.207	0.157	0.054	0.589	

Legend: G1, group pre-surgery; G2, group post-surgery; PP, peak pressure; *n*, number of ears; SD, standard deviation; Min, minimum; Max, maximum; *p*-value, * *p* ≤ 0.05.

**Table 5:** Comparison between groups in terms of amplitude of transient evoked otoacoustic emissions in peak pressure and ambient pressure conditions in both groups (paired Student’s *t*-test).

Frequency (Hz) Condition	Group	*n*	Mean	Median	SD	Min	Max	*p*-Value
Amplitude (dBSPL) 1000 PP	G1	29	14.29	13.9	5.35	4.6	29.8	* 0.015
	G2	29	12.11	11.8	6.39	−1.3	30.5	
2000 PP	G1	29	12.81	13.2	3.75	0	18.1	0.074
	G2	29	11.08	11.9	4.05	0.7	17.1	
3000 PP	G1	29	7.40	8.1	5.03	−4	18.1	0.233
	G2	29	6.61	7.3	4.60	−3.6	18	
4000 PP	G1	29	1.01	3.3	5.07	−10.7	10	0.325
	G2	29	0.33	1.8	5.67	−10.8	9.8	
5000 PP	G1	29	−7.53	−7.,1	5.12	−20	1.5	0.284
	G2	29	−8.28	−8	5.52	−17	2.1	
1000 AP	G1	29	14.10	13.8	5.60	2.5	29.2	* 0.002
	G2	29	11.79	11.6	6.30	0.7	30.5	
2000 AP	G1	29	13.21	13.4	3.80	6.9	23.1	0.511
	G2	29	17.89	12.2	38.55	−0.7	217.3	
3000 AP	G1	29	7.49	7.9	4.99	−6.4	18.2	0.095
	G2	29	6.56	7.1	4.73	−3.1	18.9	
4000 AP	G1	29	1.10	2.9	5.20	−11	10.3	0.129
	G2	29	0.12	1.5	5.93	−12.1	9.6	
5000 AP	G1	29	−7.58	−7	5.47	−19.4	2	0.534
	G2	29	−8.01	−7	5.39	−17	1.9	

Legend: G1, group pre-surgery; G2, group post-surgery; PP, peak pressure; AP, ambient pressure; *n*, number of ears; SD, standard deviation; Min, minimum; Max, maximum; *p*-value, * *p* ≤ 0.05.

**Table 6:** Comparison between groups in terms of amplitude of otoacoustic emissions by distortion product, in peak pressure and ambient pressure conditions, in the both groups (Paired student *t*-test).

Frequency (Hz) Condition	Group	*n*	Mean	Median	SD	Min	Max	*p*-Value
Amplitude (dBSPL) 500 PP	G1	30	6.55	7.15	6.98	−5.7	20.1	0.168
	G2	30	8.70	8.75	6.67	−4.3	18.2	
1000 PP	G1	30	12.80	13.55	4.72	4.7	22.3	0.557
	G2	30	13.15	13.8	4.78	2.3	23.5	
1500 PP	G1	30	15.04	15.4	5.31	4.1	25.2	0.897
	G2	30	15.11	16.5	5.30	2.9	24	
2000 PP	G1	30	12.44	12.45	4.99	2.5	22.3	0.286
	G2	30	13.08	13.25	4.38	3.8	20.6	
3000 PP	G1	30	10.24	10.35	4.47	1	19	0.662
	G2	30	10.52	11.7	4.24	2.4	17.4	
4000 PP	G1	30	11.46	12.65	4.70	1.4	19.5	0.613
	G2	30	11.09	12.45	5.35	0	19.2	
5000 PP	G1	30	10.44	11.4	5.60	−4.9	18.4	0.210
	G2	30	9.01	10.05	6.52	−5	18.5	
6000 PP	G1	30	8.08	8.35	4.73	−1.9	17.2	0.137
	G2	30	6.09	7.55	7.25	−9.4	16.8	
7000 PP	G1	30	8.43	10.25	6.21	−8.8	19	0.169
	G2	30	6.26	9.8	8.79	−10.5	16.2	
8000 PP	G1	29	−0.59	−0.3	7.93	−18.5	17.1	0.081
	G2	30	−3.60	0.95	11.39	−22.7	12.8	
9000 PP	G1	30	−2.76	−1.7	8.47	−18.7	12.4	0.054
	G2	29	−6.64	−4.3	11.37	−37.1	9.4	
10,000 PP	G1	30	−3.26	−3.4	9.07	−21.9	19.4	0.061
	G2	30	−6.71	−6.15	8.79	−22.3	8.6	
500 AP	G1	29	6.65	8.2	6.59	−11.6	18.5	0.241
	G2	29	8.72	9.9	7.21	−16.1	17.6	
1000 AP	G1	29	11.74	12.1	7.41	−6.7	24.4	0.160
	G2	29	13.66	13.9	5.31	2.8	25.1	
1500 AP	G1	29	13.91	14.7	7.66	−6.8	26.7	0.118
	G2	29	16.20	17.2	4.85	5.1	24.3	
2000 AP	G1	29	15.24	12.8	17.64	−9.6	10.9	0.631
	G2	29	13.67	13.9	4.36	3.8	21.4	
3000 AP	G1	29	10.73	10.5	6.30	−2.2	26.3	0.227
	G2	29	12.22	11.5	5.14	2.3	25.6	
4000 AP	G1	29	12.37	13.3	5.89	1.4	32.9	0.877
	G2	29	12.19	13	6.21	0.2	30.2	
5000 AP	G1	29	11.97	11.9	6.49	1.1	36.6	0.206
	G2	29	10.22	10	6.54	−6.4	21.8	
6000 AP	G1	29	9.95	8.6	7.38	0.9	41.6	0.818
	G2	29	10.87	9.8	21.11	−13.1	113.5	
7000 AP	G1	29	9.35	10.7	7.55	−8.9	29	0.314
	G2	29	7.84	12	9.23	−14.9	20	
8000 AP	G1	29	2.16	3.6	10.66	−16.9	419	* 0.048
	G2	29	−2.22	2.1	10.81	−25.5	12.8	
9000 AP	G1	29	−1.13	−2.2	10.82	−21.2	37.3	* 0.020
	G2	29	−5.98	−3.4	10.60	−28.2	9.6	
10,000 AP	G1	29	−2.26	−2.4	11.92	−29.1	2.5	0.077
	G2	29	−5.94	−3.6	9.09	−22.6	8	

Legend: G1: group pre-surgery; G2: group post- surgery; PP: peak pressure; AP: ambient pressure; *n:* number of ears; SD: standard deviation; Min: minimum; Max: maximum; *p*-value: * *p* ≤ 0.05.

**Table 7:** Comparison between groups in terms of signal/noise of otoacoustic emissions by distortion product, in peak pressure and ambient pressure conditions, in the both groups (Paired student *t*-test).

Frequency (Hz) Condition	Group	*n*	Mean	Median	SD	Min	Max	*p*-Value
S/R (dB) 500 PP	G1	30	6.33	7.4	5.43	−8.8	12.3	0.262
	G2	30	4.49	5.45	6.86	−9.3	15	
1000 PP	G1	30	16.11	14.85	5.41	7.5	27	* 0.017
	G2	30	19.79	18.95	7.81	0.3	34.2	
1500 PP	G1	30	23.14	22.25	5.21	14.7	34.3	* 0.002
	G2	30	26.90	27.25	6.75	12	41	
2000 PP	G1	30	23.11	22.6	6.01	10.2	34	* <0.001
	G2	30	27.24	27.2	5.28	16.5	40.4	
3000 PP	G1	30	24.56	23.9	5.05	14.4	35.3	* <0.001
	G2	30	28.92	29.1	4.41	19.4	36	
4000 PP	G1	30	39.87	31	56.84	15.8	339.1	0.491
	G2	30	32.85	33.4	6.45	16.9	42.3	
5000 PP	G1	29	33.72	34.4	7.18	14.1	46.3	0.291
	G2	30	35.46	37.75	6.96	19	45.1	
6000 PP	G1	30	33.68	33.65	6.00	21	48.1	0.523
	G2	30	34.78	36.7	8.04	18.1	44.3	
7000 PP	G1	30	32.03	32.25	7.38	15.9	47.6	0.610
	G2	30	33.02	35.75	8.79	16.7	42.9	
8000 PP	G1	30	24.66	23	8.24	6.4	41.9	0.582
	G2	30	23.43	24.55	12.05	−3.2	42.3	
9000 PP	G1	30	23.40	23.2	7.17	13.6	41	0.161
	G2	30	20.45	21.35	12.08	−7.1	36.3	
10,000 PP	G1	30	18.41	17.1	8.33	5.9	44.7	0.475
	G2	30	17.07	16.3	9.20	0.5	32.4	
500 AP	G1	29	6.45	8.4	6.08	−16.3	12.6	0.358
	G2	29	4.98	5.9	7.49	−16.1	19.2	
1000 AP	G1	29	15.85	16.3	5.62	−0.9	25.9	* 0.035
	G2	29	19.20	20.5	8.34	1.9	35.2	
1500 AP	G1	29	22.12	23.4	7.04	3.4	33.3	* 0.020
	G2	29	26.54	25.7	6.96	13.4	37.8	
2000 AP	G1	29	22.87	23.6	6.22	0.9	32	* <0.001
	G2	29	28.19	28.6	5.16	15.3	38.8	
3000 AP	G1	29	25.21	24.3	5.49	16.3	34.1	* <0.001
	G2	29	29.04	29.6	4.87	17.4	39.3	
4000 AP	G1	29	30.43	30.6	5.59	16.8	39.9	* 0.023
	G2	29	32.33	33.3	5.84	20.9	41.6	
5000 AP	G1	29	35.10	35.7	5.73	21.8	44.9	0.752
	G2	29	35.58	36.5	7.64	19.3	46.3	
6000 AP	G1	29	34.79	35.2	5.65	25.1	46.6	0.869
	G2	29	35.08	35.7	7.19	18.4	44.9	
7000 AP	G1	29	33.14	34.8	7.36	13.6	46.5	0.709
	G2	29	33.77	37.1	8.83	14.3	44.4	
8000 AP	G1	29	26.50	27.5	8.12	12.2	42.1	0.450
	G2	29	24.80	27.1	11.84	−3.5	41.1	
9000 AP	G1	29	23.91	25.7	7.68	9.4	37.5	0.221
	G2	29	21.31	22.8	11.83	−4.9	36.9	
10,000 AP	G1	29	17.51	16.2	10.25	−9.3	43.1	0.693
	G2	29	16.73	19.3	9.95	0.3	31.1	

Legend: G1: group pre-surgery; G2: group post- surgery; PP: peak pressure; AP: ambient pressure; *n*: number of ears; SD: standard deviation; Min: minimum; Max: maximum; *p*-value: * *p* ≤ 0.05.

## Data Availability

The raw data supporting the conclusions of this article will be made available by the authors on request due to privacy concerns. The data in tables are deposited in the UNICAMP research data repository (REDU) and can be accessed via the link: https://doi.org/10.25824/redu/VYLYMD, accessed on 29 May 2024.
